# Influence of Buffer-Protected Sodium Butyrate Supplementation in the Diet of *Penaeus vannamei*

**DOI:** 10.1155/anu/5578544

**Published:** 2025-08-18

**Authors:** Maria Érica da Silva Oliveira, Joice Teixeira Souza, Ana Elidarly Cunha, Vanessa Maria Freitas Silva, Mário Augusto Monteiro Silva, Daniela Nomura Varandas, Andreia Vilas Boas, Ana Louise Toledo, Marisela Arturo Schaan, Javier Sánchez, Beatriz Saldaña, Moacir Franco de Oliveira, Matheus Ramalho de Lima

**Affiliations:** ^1^Animal Science, Universidade Federal Rural do Semi Árido, Campus Mossoró, Mossoró 59625-000, Brazil; ^2^Vidara do Brasil, Jundiaí 13211-620, Brazil; ^3^Deltavit CCPA Group, ZA du Bois de Teillay, Janzé 35150, France; ^4^Novation 2002, SL., CCPA Group, Arcos de Jalón 42250, Spain

**Keywords:** health, organic acid, physiological changes, sodium butyrate

## Abstract

This study aimed to determine the optimal inclusion level of protected sodium butyrate (PSB) in diets for *Penaeus vannamei* during the postlarvae to grow-out phase. A completely randomized design was used with four dietary treatments: 0, 2, 4, and 8 kg/t of PSB (54% sodium butyrate). The PSB product (Novation SL 2002, Spain) contains 54% sodium butyrate protected by a physical and chemical matrix based on buffer salts. Growth performance, feed efficiency, survival, histological parameters of the intestine and hepatopancreas, and meat quality were evaluated. The results demonstrated that PSB supplementation significantly improved growth performance, feed conversion ratio (FCR), survival, and gut histomorphology, as well as enhanced meat production and quality. Based on the growth and feed efficiency responses, the optimal PSB inclusion level is recommended between 4 and 6 kg/t. Therefore, PSB is an effective dietary additive to improve productivity, gut health, and product quality in *P. vannamei* farming.

## 1. Introduction

The industrial farming of *Penaeus vannamei* faces numerous challenges, including diseases, poor growth performance, and stress caused by environmental conditions. These factors negatively impact shrimp productivity and survival rates in aquaculture systems, often leading to increased use of antibiotics. However, this practice presents significant risks as antibiotic residues can accumulate in the environment, causing pollution and biomagnification in food chains [1]. In this context, enhancing shrimp health and growth while reducing dependance on antibiotics has become a top priority in the industry. Sustainable alternatives, including vaccines, probiotics, and natural antimicrobials, offer promising solutions to address these challenges. These strategies not only mitigate the risks associated with antimicrobial resistance, but also promote environmental sustainability, ensuring the long-term viability of shrimp farming [1–3].

In aquaculture, some studies show that the use of butyric acid offers several possibilities, with results indicating an increase in growth, a reduction in stress, an enhancement of digestive capacity and function, and higher survival rates [4–8]. The bioavailability of butyric acid varies significantly depending on its form, as demonstrated by research across different contexts. Butyric acid is utilized in various forms, including salts, coated salts, and glycerides, each with a distinct bioavailability and effectiveness profile [9].

Based on this, protected sodium butyrate (PSB) has been identified as a promising dietary supplement for *P. vannamei*, offering several health benefits, particularly in terms of modulating gut microbiota and enhancing immune responses. While PSB shows significant potential in aquaculture, it is important to consider its availability and cost-effectiveness as a dietary additive, particularly in large-scale farming operations. The effect of PBS on *P. vannamei*, commonly known as white-leg shrimp, has been extensively studied, revealing its impact on growth performance, intestinal microbiota, immunological parameters, and survival rates. While other forms of butyric acid, such as poly-*β*-hydroxybutyrate (PHB) and tributyrin, have also been studied for their impact on shrimp health, PSB has demonstrated efficacy in promoting intestinal health and modulating immune functions in *P. vannamei* [3, 4, 6]. PHB and tributyrin, although having shown some promise in enhancing intestinal function and microbial balance, are not the primary focus of this research.

Research indicates that sodium butyrate supplementation in shrimp diets can enhance survival and productivity by modulating the immune system and reducing the concentration of pathogenic bacteria in the gut, thereby improving overall health and resistance to diseases, such as *Vibrio alginolyticus* [10, 11]. Research has demonstrated that sodium butyrate has the potential to enhance intestinal health and morphology, which are essential for nutrient absorption and overall growth performance in aquatic species [3, 4, 6, 8, 12, 13]. For example, dietary supplementation with sodium butyrate was found to mitigate growth reduction and enteritis in fish by promoting the activities of intestinal digestive enzymes, decreasing mucosal permeability, and attenuating intestinal inflammatory responses. These findings may also be applicable to shrimp species, such as *P. vannamei* [14].

Sodium butyrate supplementation has been linked to enhanced feed efficiency and growth performance in shrimp. This was evident in a study conducted by [5, 15], where shrimp fed diets fortified with sodium butyrate demonstrated an increased final weight compared to the control groups. Moreover, sodium butyrate has been shown to elevate the populations of beneficial lactic acid bacteria in the intestines, while having no impact on the counts of *Vibrio sp*. or total heterotrophic bacteria [8]. These findings suggest that sodium butyrate plays a vital role in promoting a well-functioning gut microbiome, as highlighted in a study by [15].

PSB has the potential to enhance the effectiveness of the immune system in *P. vannamei*, thereby becoming a valuable tool for managing health in shrimp farming. However, it is important to note that the benefits of PSB are specific and distinct from those of other organic acid salts. While PSB offers advantages in terms of gut health and immune response, other salts may not yield the same level of benefit in these areas. An example of this can be seen in a study conducted by [15], where the effects of different forms of artificially salinized water on growth parameters and bacterial counts in *P. vannamei* juveniles did not directly correlate with the specific benefits attributed to PSB. This highlights the unique role that PSB plays in aquaculture nutrition. In summary, PSB serves as a valuable dietary supplement for *P. vannamei*, bolstering survival, growth, and health by specifically modulating the gut microbiota and enhancing immune responses. These benefits distinguish PSB from other organic acid salts, as demonstrated in studies by [14–18]. Furthermore, a study by [13] investigating the effects of butyric acid on juvenile Pacific shrimp (*P. vannamei*) revealed that dietary inclusion of butyric acid significantly improved survival rates during heat stress. Specifically, shrimp supplemented with 1.5% of this acid exhibited no mortality, indicating a protective effect against heat-induced stress. In addition, this supplementation led to improved immune responses, as evidenced by increased activities of alkaline phosphatase (AKP) and acid phosphatase (ACP), as well as enhanced antioxidant enzyme activity, such as superoxide dismutase (SOD).

Contrastingly, research on various organic acids and their salts, such as benzoic acid, has demonstrated negative effects on the well-being of shrimp, indicating that not all organic additives have advantageous effects on *P. vannamei* [18, 19]. This emphasizes the distinctiveness of sodium butyrate's beneficial role in shrimp aquaculture, setting it apart from other organic acid supplements. However, it is important to note that while sodium butyrate offers multiple advantages, its efficacy can vary based on the concentration utilized and the specific parameters of health or growth being assessed [11]. In summary, sodium butyrate serves as a valuable dietary supplement for *P. vannamei*, fostering improved survival, growth, and well-being by modulating the gut microbiota and enhancing immune responses, although its benefits are specific and divergent from other organic acid salts.

The aim was to evaluate the effect of three levels of buffer-PSB in the diet of *P. vannamei* shrimp during the nursery and grow-out phases.

## 2. Materials and Methods

### 2.1. Study Location and Experimental Design

This study was conducted at the Animal Sciences Department of the Federal University of Rural Semi-Arid Region (UFERSA), located in Mossoro, Rio Grande do Norte, Brazil. The water used for cultivation was sourced from an artesian well, characterized by low salinity levels (3 ppt).

A completely randomized design was employed in this experiment, with treatments including four levels of PSB supplementation (54% sodium butyrate), at concentrations of 0, 2, 4, and 8 kg/t. Prior to experimental stocking, the quality of postlarvae was evaluated under three environmental stressors: salinity, temperature, and nitrite lethal dose (DL70). The postlarvae used in the study (JH Pós-Larvas, Aracati, CE, Brazil; SPF larvae) were confirmed to be of high quality. PSB supplementation (Novation SL 2002, Spain) with 54% sodium butyrate encapsulated in a physical and chemical matrix of buffer salts was used in this study.

After confirming the postlarvae quality, the animals were acclimated in a 20 m^3^ geomembrane tank for a period of 19 days (PL11 to PL30, 10 PL/g). Following the acclimatization and growth period, the animals were weighed, and the average weight was calculated to organize homogeneous batches for experimental purposes. The initial average weight of the postlarvae was 93.18 ± 1.617 mg, with an initial mean length of 25.06 ± 0.414 mm.

### 2.2. Experimental Diets

The experimental diets were formulated to provide 37% crude protein, 1.3% fat, 0.3% fiber, 1.4% ash, and 12.35% fish meal (FM), supplemented with additives according to the treatment levels. The feed particles ranged from 600 to 800 µm (Exteex 500x, São Paulo, Brazil). The treatments were organized with increasing levels of PSB supplementation (0, 2, 4, and 8 kg/t). The PSB was incorporated into the feed using 1% soybean oil as a diluent, which was sprayed onto the feed and then dried to achieve the desired composition. Each treatment was replicated four times, except for the control group, which was replicated eight times, with 160 shrimp per replicate during the nursery phase and 75 shrimp per replicate during the grow-out phase.

Feeding was conducted using pellets ranging from 1.5 to 2 mm in size. Feeding trays (47.5 cm in diameter, 8.7 cm in height, black color) were installed in each replicate for the experimental units. After 1 h and 30 min, any remaining feed was collected, dried, and weighed to calculate the feed conversion ratio (FCR). The diets were formulated to meet the nutritional requirements of the shrimp at each experimental phase [20] (Table 1).

### 2.3. Shrimp in Experimental Phases: Nursery and Grow-Out Phases

During the nursery phase, postlarvae (PL30) were housed in blue polypropylene tanks with a capacity of 500 L (0.5 m^3^), each equipped with independent aeration and water-supply systems. Each experimental unit contained a feeding tray (47.5 cm in diameter, 8.7 cm in height, black color).

In the grow-out phase, 75 juveniles from each replicate per treatment, previously harvested from the nursery phase, were weighed and transferred to hapas made of nylon multifilament-coated PVC mesh net pens, with a mesh size of 5 mm and a useful volume of 1 m^3^.

### 2.4. Water Parameters

During the shrimp experiment, various dietary regimens were administered while multiple aquatic parameters were systematically observed every 3 days. The parameters included pH, measured using a pH meter (Model Hanna Instruments HI 98127, manufactured by Hanna Instruments, Woonsocket, RI, USA); conductivity (µS/cm), measured using a conductivity meter (Model YSI ProDSS, manufactured by YSI, Yellow Springs, OH, USA); salinity (parts per million [ppm]), measured using a salinity refractometer (Model ATAGO PR-32α, manufactured by ATAGO Co., Ltd., Tokyo, Japan); temperature (°C), documented using a thermometer (Model Thermo Scientific Traceable, manufactured by Thermo Fisher Scientific, Waltham, MA, USA); dissolved oxygen (DO [mg/L]), measured using a DO meter (Model YSI ProDSS, manufactured by YSI, Yellow Springs, Ohio, USA); total dissolved solids (TDS [mg/L]), quantified using a TDS meter (Model HM Digital TDS-4, manufactured by HM Digital, Los Angeles, CA, USA); ammonia (mg/L), quantified using an ammonia test kit (Model LaMotte 5887, manufactured by LaMotte Company, Chestertown, MD, USA); and nitrite (mg/L), evaluated using a nitrite test kit (Model API 5-in-1 Freshwater Master Test Kit, manufactured by Mars Fish care, Chalfont, PA, USA). Details of these measurements are presented in Table 2.

### 2.5. Performance Data

In the nursery phase, performance parameters were assessed after 35 days by capturing, counting, and weighing the shrimp to determine live weight (g/shrimp), weight gain (g/shrimp), feed intake (g/shrimp), and FCR (g/g). During the grow-out phase, shrimp were captured from 20% of the initial biomass every two weeks for weighing and for determining the same performance parameters assessed during the nursery phase. At the end of the 90-day cultivation period, all shrimp were collected, weighed, and counted to obtain survival data, which were calculated as the difference between the number of shrimps at the beginning and the end of the experimental period, with all mortalities recorded. Additionally, at the conclusion of the experiment, 30 shrimp per treatment were selected to measure length and assess individual weight. The head and carapace were removed to calculate the headless shrimp yield (shrimp [%]) and fillet yield (meat [%]).

### 2.6. Hematological Analysis

A total of 300 µL of anticoagulant solution was added to the collection syringe. The ventral sinus of the shrimp was disinfected with 70% alcohol, and the shrimp was positioned with its ventral side facing upward. Hemolymph (100 µL) was extracted from the area between the first abdominal segment and the cephalothorax. The hemolymph was thoroughly mixed with the anticoagulant solution and transferred to a labeled Eppendorf tube. The mixture was stored in a refrigerator until further processing.

Prior to counting, 20 µL of rose bengal dye were added to the sample and homogenized. After a 20 min incubation period, the homogenized material was transferred to a clean Neubauer chamber until the counting area was filled. The sample was allowed to settle for 2 min before hemocyte quantification. The counting area (1 mm^2^, height = 0.1 mm) was divided into 25 subareas, with 5 subareas examined diagonally. The total number of cells counted across these subareas was used in the following formula to calculate the hemocyte concentration. For this analysis, the dilution factor was determined as 4.2 = (100 μL + 300 μL + 20 μL)/100 μL.

This method provided a reliable assessment of shrimp health status and was applied for preliminary diagnoses in both laboratory and field settings.

During each sampling event, five specimens were randomly selected at 7:00 a.m. and maintained in aerated conditions. Macroscopic analysis included evaluating the morphology and pigmentation of the antennae, pleopods, and uropods. Microscopic analysis focused on the condition of tissues including the gills, cephalothorax, epipodite, hepatopancreas, and intestinal tract. The severity of observed changes or injuries was scored on a scale from 0 (no observable change) to 4 (high degree of alteration).

Additional assessments included the following:

• Antennae: Roughness and pigmentation.
• Cephalothorax: Examination for melanization, necrosis, and calcium deposits.
• Gills: Evaluation of coloration changes, melanization, necrosis, and protozoan presence (e.g., *Zoothamnium sp*., *Epistylis sp*., *Acineta*, *Ascophris*, and *Bodo sp*.).
• Hepatopancreas: Inspection for atrophy, hypertrophy, and signs of necrosis.
• Epipodite: Microscopic evaluation for melanization, necrosis, and other alterations.
• Intestinal tract: Evaluation for hyperplasia, gregarines, and saprophagia.

### 2.7. Tissue Histology—Intestine and Hepatopancreas

Histological samples were collected from 15 shrimp per treatment for conventional light microscopy analysis. Tissue samples (~0.5 cm) were fixed in 4% paraformaldehyde buffered with 0.1 M sodium phosphate (pH 7.4) at 4°C. After dehydration and clearing, the samples were embedded in paraffin (Synt granulated, melting point 58–62°C). Samples were immersed in two paraffin baths, first at 60°C for overnight impregnation and then at 60°C for 1 h to ensure complete paraffin infiltration.

Following embedding, tissue blocks were sectioned at 5–7 µm using a LEICA RM 2125 RT microtome, and the sections were placed on glass slides and dried at 60°C for a maximum of 6 h. The slides were deparaffinized in two xylene baths (10 min each), rehydrated in decreasing alcohol concentrations (100%, 95%, and 70%), and washed in running water. Staining was performed using hematoxylin–eosin (HE) to highlight tissue structures [21].

The sections were analyzed under a light microscope (LEICA DM 500 HD, Wetzlar, Germany), and relevant images were captured through photomicrography. Histological measurements were recorded using a micrometric eyepiece graduated in millimeters. The data were analyzed using ImageJ2 (1.54 k, 2024, USA) to compare differences across treatments.

### 2.8. Sample Collection

In both the nursery and grow-out phases, a total of 20% of the initial biomass was sampled biweekly, with all animals collected at the end of the 90-day period for final analysis.

For hemolymph collection, 15 shrimp per experimental unit were selected and a sterile syringe was used to extract the hemolymph from the cephalothorax.

For histological analysis, 15 shrimp per treatment were dissected at the end of the experiment to collect tissue samples from the intestine and hepatopancreas.

Regarding performance data, shrimp were weighed and counted throughout both phases to determine growth, feed intake, and survival rates.

### 2.9. Statistical Analysis

Data normality was first assessed using the Shapiro–Wilk test. Once normality was confirmed, an analysis of variance (ANOVA) was conducted. Survival data (%) were transformed using the arcsine square root transformation before statistical analysis. (“Toxicity of Benzotriazole and Benzotriazole Derivatives to Three …”).

To compare data across the nursery and grow-out phases, polynomial regression models were applied to the PSB levels over time, with time-dependent regressions considered for all treatments. The most appropriate polynomial order for each model was determined through ANOVA and the coefficient of determination (*R*^2^).

Statistical analyses were performed using R software (v. 2024.12.0), and results were considered statistically significant when the *p*-value was less than 0.05.

## 3. Results

### 3.1. Performance Data

**Figure 1:**
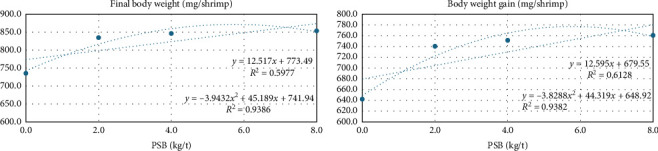
Final body weight of PL65 (FBW, mg), body weight gain (BWG, mg/shrimp) during the 35 days, from PL30 to PL65 (nursery phase), of shrimp fed increasing levels of protected sodium butyrate (PSB).

There was a linear effect with the dose increase on length (*p*=0.0041), weight per unit length (mg/mm, *p*=0.005), body weight (*p*=0.004), weight gain (*p*=0.004), final-to-initial live weight ratio (*p*=0.014), survival (*p* < 0.001), and improved feed conversion ratio (*p*=0.003) as shown in Table 3 (nursery phase). Additionally, there was a significant quadratic effect on final body weight (FBW, *p*=0.044| FBW = 741.94 + 45.189*x* − 3.9432*x*^2^; *R*^2^ = 0.9386), and body weight gain (BWG, *p*=0.053| BWG = 648.92 + 44.319*x* − 3.8288*x*^2^; *R*^2^ = 0.9382), with a value based on the equation of 5.73 and 5.78 kg/t, respectively, of PSB in the diet (Figure 1).

**Figure 2:**
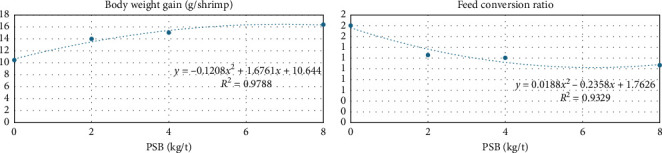
Body weight gain (BWG [g/shrimp]) and feed conversion ratio (FCR [g/g]) during the 80 days after nursery phase (grow-out phase) of shrimp fed increasing levels of protected sodium butyrate (PSB).

As shown in Table 4, during the grow-out phase, the increase in the level of PSB significantly increased the weight gain and its uniformity, the length of the shrimp, and the body weight gain, and improved the FCR, allowing for shrimp with a higher weight per unit of size, a higher final weight to initial weight ratio, and survival at the growth phase. These data show a result that allows us to recommend an optimal dose of PSB by a BWG of 6.94 kg/t (BWG = 0.1208*x*^2^ + 1.6761*x* + 10.644 | *R*^2^ = 0.98), and an FCR of 6.27 kg/t (FCR = 0.0188*x*^2^ − 0.2358*x* + 1.7626| *R*^2^ = 0.93), Figure 2.

**Figure 3:**
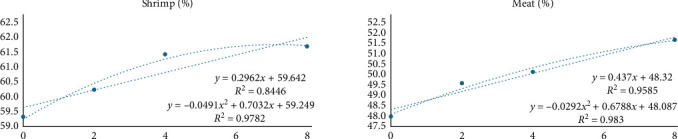
The relative weight of the shrimp without head (shrimp[%]), and without all shells (meat[%]) of shrimp meat fed with levels of protected sodium butyrate (PSB).

PSB supplementation linearly increased the yield of shrimp (head-off) and the yield of shrimp fillet (head-off and shell-off) (Table 5, and Figure 3). The improvement in yield shows that PSB allowed not only a higher final weight of the animals but also an important increase in meat production.

### 3.2. Hematological Analysis

**Figure 4:**
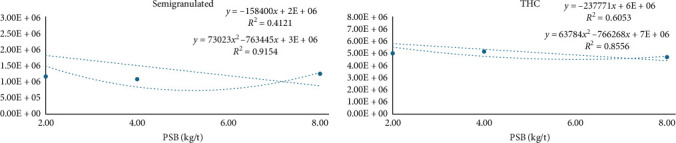
Semigranulated cells and the total hemocyte count (THC).

Table 6 presents the cell types and total hemocyte count (THC) for shrimp on varying PSB diets. Granular cells function in the immune response, while hyaline cells facilitate healing, revealing no dietary PSB impact. The THC results mirrored those of granular cells, with a decreased linear and quadratic influence (point: 6.00 kg/t of PSB, Figure 4). PSB enhanced the semigranulated cells (*p*=0.0324, linear and quadratic, point: 5.2 kg/t of PSB, Figure 4). These cells exhibit dual characteristics and can differentiate into granular or hyaline types.

### 3.3. Tissue Histology

**Figure 5:**
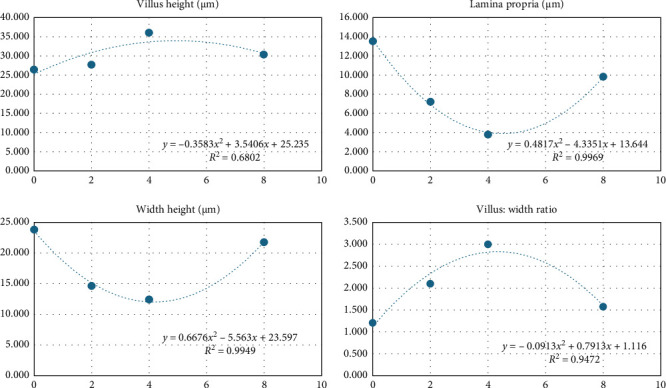
Shrimp intestine histomorphometry fed with levels of protected sodium butyrate (PSB).

PSB influenced the height of the villi and the other variables in a quadratic manner, with the levels determined in Table 7. In this case, increased villus height indicates a greater intestinal surface area, which can lead to improved nutrient absorption from the diet. In the table, villus height appears to be the highest at 4 kg/t PSB and the lowest at the control (0 kg/t PSB), with an optimal dose of 4.94 kg/t of PSB, Figure 5.

Regarding width height, a wider villus base could provide more support for the villus structure and potentially a greater surface area for absorption, and the optimal dose of this was 4.16 kg/t of PSB, Figure 5.A thicker lamina propria might indicate increased blood vessels supplying the intestine, based on the supplementation of PSB in the diet. The lamina propria shows an optimal dose of 4.49 kg/t of PSB, Figure 5. A higher H:W ratio typically indicates tall and slender villi, which may be more efficient for nutrient absorption compared to short and stubby villi. The table shows the H:W ratio is the highest at 4 kg/t PSB, or specifically 4.33 kg/t potentially aligning with the highest villus height at this dose, Figure 5.

The observed influence of PSB on villus height, width, and lamina propria thickness suggests a significant modulation of intestinal morphology, potentially impacting nutrient absorption efficiency (Figure 3). Increased villus height, as seen at 4 kg/t PSB, implies a larger intestinal surface area, facilitating enhanced nutrient absorption from the diet. Similarly, wider villi bases, observed at 4.16 kg/t PSB, may provide additional structural support and surface area for absorption. The thicker lamina propria observed at 4.49 kg/t PSB suggests increased vascularization, potentially improving nutrient delivery to the intestine. The higher H:W ratio at 4 kg/t PSB indicates taller and slender villi, which are typically more efficient for nutrient absorption.

**Figure 6:**
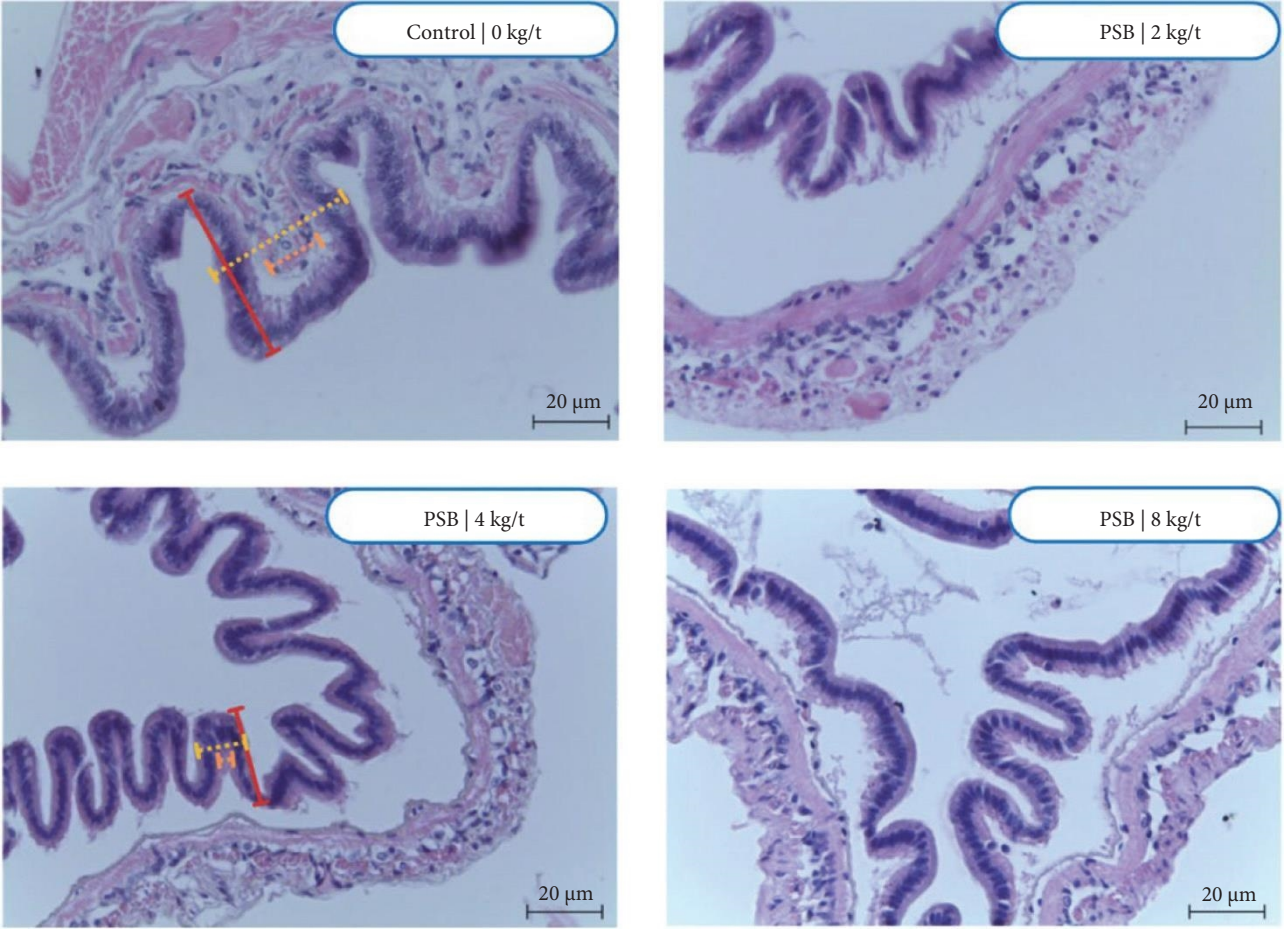
Histological images of the intestines of shrimp fed with protected sodium butyrate (PSB). Stained with hematoxylin-eosin.

The intestine of the control group, which did not receive PSB, shows a less defined structure with irregular shapes, Figure 6. At a lower dose of 2 kg/t PSB, the intestine appears more structured compared to the control, suggesting that even low levels of PSB influence intestinal structure. At higher doses, particularly 8 kg/t, the intestine becomes more well-defined, indicating a strengthening or modifying effect of PSB on the intestinal structure.

**Figure 7:**
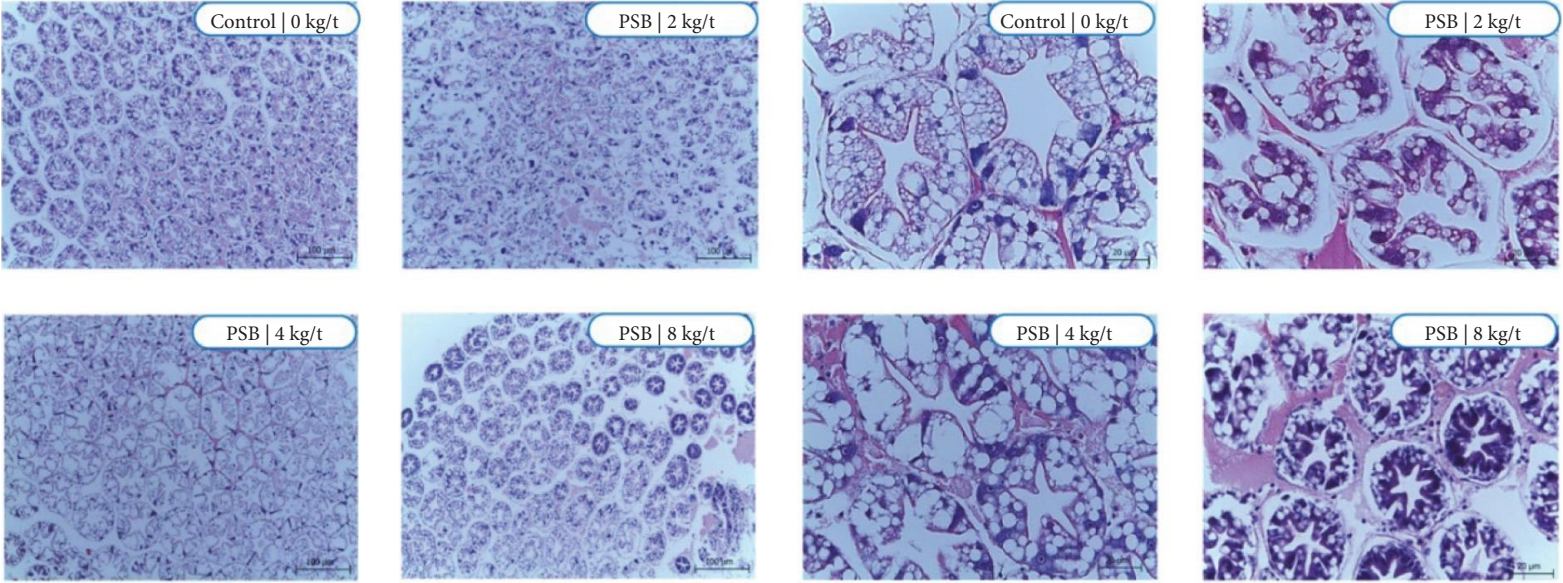
Histological images of the hepatopancreas of shrimp fed with protected sodium butyrate (PSB). Stained with hematoxylin–eosin, 100 and 20 µm.

Similarly, the hepatopancreas in the control group shows a dense aggregation of cellular structures (Figure 7). However, no significant changes were observed in the lipid reservoir across PSB levels. At the highest dose (8 kg/t), an increase in cellular density and glycogen storage within B cells was noted, suggesting enhanced energy metabolism and improved resilience to environmental stress.

The supplementation of PSB seems to have influenced the thickening of the interstitial tissue, with a potential increase in phagocytosis. The thickening of this tissue could potentially enhance these exchanges, leading to improved cellular function and overall health. An increase in phagocytosis suggests an enhanced immune response, which could help the body to better defend against infections and diseases. By potentially enhancing, both the structure of the interstitial tissue and the process of phagocytosis, PSB supplementation could contribute to improved cellular function and immune response.

## 4. Discussion

### 4.1. Performance and Yields

The application of PSB as a dietary supplement has garnered increasing interest in aquaculture due to its potential to enhance growth performance, survival rates, and overall health in shrimp. This study aims to expand on previous research, which has demonstrated variable outcomes based on dosage, formulation, and cultivation conditions. For example, a study by [22] observed a 9% increase in weight and a 3% improvement in the survival rate for *Penaeus monodon* when sodium butyrate was encapsulated in vegetable oil at a 0.1% concentration. Similarly, studies using concentrations of 0.5%, 1%, and 2% in *P. vannamei* reported significant improvements in live weight and survival rates, especially at the highest concentration [5]. However, other research has shown that when 2 kg/t of sodium butyrate was incorporated into a biofloc system, no growth improvements were noted; yet, survival rates increased, leading to higher overall productivity [11]. These findings emphasize that the efficacy of sodium butyrate can vary depending on environmental conditions, dosage, and species.

In contrast to the promising effects of sodium butyrate, some organic acids, such as benzoic acid, have been shown to negatively impact the growth and health of *P. vannamei*, highlighting that not all feed additives provide benefits under all conditions [23]. Recent studies have explored the microencapsulation of organic acids and their salts, demonstrating that they can improve shrimp performance, digestive enzyme activity, immunity, and pathogen resistance, which may reduce the need for antibiotics in aquaculture systems [24]. These findings illustrate the diverse approaches available for enhancing shrimp productivity, which include diet modifications, feed additives, and environmental management strategies. However, the effectiveness of these approaches depends on factors, such as the shrimp species, the specific aquaculture system, and the formulation of the diet or biofloc system.

Our study results align with those of previous research, which show that PSB supplementation improves shrimp growth performance. We observed that the optimal supplementation levels during the nursery phase were 5.73 and 5.78 kg/t of PSB, which led to the highest final weight and weight gain. In the grow-out phase, supplementation levels of 6.94 and 6.27 kg/t of PSB yielded the best weight gain and feed conversion, respectively. This suggests that PSB supplementation may enhance the productivity of the shrimp *P. vannamei*.

### 4.2. Histology

Histological evaluations revealed significant structural changes in the intestines of shrimp receiving PSB supplementation. At elevated levels of PSB, particularly at 8 kg/t, the intestine showed a more distinct and well-organized morphology compared to the control group, which had a less defined structure. These findings suggest that PSB supplementation can positively influence intestinal morphology, improving the overall intestinal health of shrimp. This observation is important, as a healthy intestine is crucial for nutrient absorption, pathogen resistance, and overall shrimp health.

In addition to improvements in intestinal structure, our results also showed increased cellular density and glycogen storage at the 8 kg/t dose of PSB. Glycogen is an essential energy source that helps to maintain metabolic balance, particularly under stress. This finding is consistent with previous studies that have demonstrated the positive impact of dietary supplements on growth performance and lipid metabolism in shrimp, such as glycerol monolaurate [25] and cinnamaldehyde [26]. However, excessive PSB supplementation could potentially lead to cellular damage and metabolic imbalances, particularly under stress conditions or pathogenic challenges, such as infection by *Enterocytozoon hepatopenaei* [27].

The observed improvements in both intestinal and hepatopancreatic structures emphasize the multifaceted benefits of PSB supplementation. These changes contribute to the overall health and performance of shrimp by improving energy metabolism, stress resilience, and immune function. However, the results also highlight the necessity of optimizing dietary strategies to ensure shrimp health, as excessive supplementation could lead to negative outcomes.

Sodium butyrate, a short-chain fatty acid produced by gut microbiota, has been shown to exert multiple beneficial effects on hepatopancreas health. It enhances lipid metabolism in hepatocytes, alleviates hepatic steatosis, and regulates genes associated with fatty acid oxidation [28]. Moreover, sodium butyrate inhibits aerobic glycolysis in hepatocellular carcinoma cells, inducing apoptosis and suppressing cellular proliferation, suggesting its potential as a therapeutic agent in liver diseases [19, 29]. Additionally, sodium butyrate's anti-inflammatory and liver-protective effects further underscore its therapeutic potential for liver-related conditions. These properties highlight the importance of sodium butyrate in promoting hepatopancreas health in shrimp and improving overall aquaculture productivity.

In summary, supplementation with PSB showed promising results in enhancing the growth, survival, and health of *P. vannamei*, although the effects varied depending on dosage, formulation, and environmental conditions. Our study confirmed that PSB supplementation significantly improved weight gain and the FCR, with optimal dosages ranging from 5.73 to 6.94 kg/t during the nursery and grow-out phases. Hemolymph analysis revealed improvements in immune function, including increased THC and immune markers, suggesting enhanced immunity in the shrimp. Additionally, PSB promoted intestinal structure and glycogen storage, potentially increasing stress resistance.

### 4.3. Hematology

The hematological analyses in our study indicated that PSB supplementation had a significant impact on shrimp hemolymph, with optimal concentrations calculated at 5.2 and 6.0 kg/t, respectively, based on the influence of PSB on cellular components. Our results suggest that PSB supplementation is dose-dependent, with higher concentrations yielding more pronounced effects on hemolymph composition. Notably, at the 4.0 kg/t level of PSB, we observed significant improvements in hemocyte levels, possibly due to PSB's role in enhancing the intestinal barrier, which may reduce pathogen translocation into the hemolymph. However, the impact of PSB on overall immunological responses remains somewhat unclear and may depend on dosage.

Principal component analysis (PCA) revealed notable similarities among the PSB-supplemented treatments, with a clear distinction from the control diet (0 kg/t PSB). The analysis indicated a reciprocal relationship between hyaline and semigranulated cells and a balanced status of THC. These results suggest that PSB supplementation has a significant effect on shrimp hemolymph that might not be fully captured by univariate analysis alone. Overall, our findings highlight that PSB supplementation positively impacts the immune response of *P. vannamei*, which may enhance the shrimp's overall health and resilience.

While previous studies, such as [5], did not report significant effects on hemolymph parameters with sodium butyrate supplementation, other studies, like [11], found improvements in total and granular hemocyte counts, as well as elevated hyaline cell levels, indicating enhanced immune activity. Similarly, the dietary inclusion of PSB improved key immune parameters, such as THC, phenoloxidase activity, and lysozyme levels, which are critical for shrimp immunity. These findings suggest that PSB supplementation can enhance immune responses in *P. vannamei*, potentially improving overall health and productivity.

Additionally, butyrate disrupts lipid rafts in cell membranes, reducing the invasion of enteric pathogens, such as *Shigella* and *Salmonella*, and promotes a cytokine balance that favors anti-inflammatory responses [30]. In fish, sodium butyrate supplementation enhances immune indices, bactericidal activity, and phagocytosis, significantly reducing mortality rates following *Pseudomonas aeruginosa* infection. Furthermore, butyrate modulates the expression of toll-like receptors (TLRs) and their downstream signaling pathways, which are pivotal in recognizing pathogens and initiating immune responses [31]. It also inhibits the NF-*κ*B signaling pathway, reducing the production of pro-inflammatory cytokines and enhancing the expression of cathelicidin (LL37), which is vital for combating *Mycobacterium bovis* infection [32]. Collectively, these studies underscore the potential of sodium butyrate as a therapeutic agent that enhances host defense mechanisms, offering a promising alternative to traditional antibiotics in managing infections and maintaining immune homeostasis.

A preprint has previously been published [33].

## 5. Conclusions

Supplementing PSB in the diets of *Penaeus vannamei* from postlarval to grow-out stages result in significant enhancements in shrimp health, histological development, and overall performance. The study demonstrated that even at lower dosages, PSB positively influenced intestinal structure, with more pronounced effects observed at higher concentrations. These improvements in intestinal morphology, coupled with enhanced cellular density and glycogen storage in the hepatopancreas, suggest that PSB supplementation plays a critical role in optimizing energy metabolism and stress resilience in shrimp.

Based on these results, the recommended dietary inclusion level of PSB is between 4 and 6 kg per ton of feed, providing the best balance between biological effectiveness and practical applicability. Consequently, PSB can contribute to improved growth, survival, and disease resistance, offering a potential strategy to enhance the sustainability and efficiency of shrimp farming.

## Figures and Tables

**Table 1:** Dietary and nutritional composition of the basal diet that received the PSB doses.

Items (%)	Basal diet (%)
Soybean meal (45% CP)	41.445
Corn	31.420
Fish meal (51.18% CP)	11.276
Fish oil	4.500
Artemia biomass^a^ (50% CP)	4.075
Soy lecithin	2.500
Salt	1.251
Potassium	1.200
Premix^b^	1.000
Vitamin C	0.500
L-Lysine HCl	0.3795
DL-Methionine	0.2575
Adsorbent	0.1000
Binder	0.0350
Antioxidant	0.0300
Antifungal	0.0300
Control diet (%)	—
PSB (%)	—
Total	100.0
Humidity (%)	10.237
Dry matter (%)	83.763
Ash (%)	7.513
Total digestible nutrients (%)	56.208
Crude energy (kcal/kg)	4200.0
Crude protein (%)	35.0
Fiber (%)	4.768
ADF (%)	6.983
NDF (%)	10.499
Methionine (%)	0.800
Met + Cis (%)	1.300
Lysine (%)	2.100
Threonine (%)	1.400
Tryptophan (%)	0.300
Arginine (%)	1.900
Isoleucine (%)	1.000
Valine (%)	1.610
Fat (%)	5.676
Linoleic acid (%)	1.260
Linolenic acid (%)	1.200
Arachidonic acid (%)	0.688
Calcium (%)	1.000
Phosphorus (%)	0.733
Sodium (%)	0.350
Potassium (%)	1.316
Chloride (%)	1.002

^a^BioArtemia, Brazil (50% CP, 9% fat, 2% fiber, and 15% ash).

^b^Vitamin A at 1,400,000 IU/kg, vitamin D3 at 800,000 IU/kg, vitamin E at 30,000 IU/kg, vitamin K at 6000 mg/kg, vitamin B1 at 8000 mg/kg, vitamin B2 at 8000 mg/kg, vitamin B6 at 8000 mg/kg, vitamin B12 at 10,000 mcg/kg, biotin at 200 mg/kg, folic acid at 2000 mg/kg, pantothenic acid at 20,000 mg/kg, niacin at 20,000 mg/kg, choline at 20,000 mg/kg, vitamin C at 30,000 mg/kg, inositol at 20,000 mg/kg, copper at 14,000 mg/kg, cobalt at 2000 mg/kg, iron at 6000 mg/kg, iodine at 160 mg/kg, manganese at 8000 mg/kg, zinc at 40,000 mg/kg, selenium at 60 mg/kg, inorganic selenium at 60 mg/kg, chromium at 2 mg/kg, and BHT at 100 mg/kg.

**Table 2:** Water parameters during the nursey and growth phases of cultivation of shrimp fed with protected sodium butyrate (PSB).

Nursey phase								
Treatment PSB (kg/t)	pH	Conductivity (µS/cm)	Salinity (ppm)	Temperature (°C)	DO (mg/L)	TDS (mg/L)	Ammonia (mg/L)	Nitrite (mg/L)
PSB|0 kg/t	8.01 ± 0.19	4.41 ± 0.04	2.74 ± 0.16	29.71 ± 0.92	6.73 ± 0.21	2.21 ± 0.81	0.025 ± 0.002	0.04 ± 0.001
PSB|2 kg/t	8.24 ± 0.13	4.41 ± 0.02	2.42 ± 0.14	29.43 ± 0.96	7.00 ± 0.23	2.21 ± 0.84	0.010 ± 0.002	0.02 ± 0.001
PSB|4 kg/t	8.24 ± 0.14	4.43 ± 0.01	2.43 ± 0.11	29.18 ± 0.89	7.00 ± 0.20	2.22 ± 0.87	0.010 ± 0.002	0.04 ± 0.001
PSB|8 kg/t	8.10 ± 0.15	4.42 ± 0.03	2.42 ± 0.21	29.85 ± 0.91	7.35 ± 0.23	2.21 ± 0.85	0.000 ± 0.002	0.02 ± 0.001

**Growth phase**								
**Treatment** **PSB (kg/t)**	**pH**	**Conductivity** **(µS/cm)**	**Salinity** **(ppm)**	**Temperature** **(°C)**	**DO** **(mg/L)**	**TDS** **(mg/L)**	**Ammonia** **(mg/L)**	**Nitrite** **(mg/L)**

PSB|0 kg/t	7.45 ± 0.32	5.34 ± 0.35	2.98 ± 0.34	28.65 ± 0.73	6.32 ± 0.32	3.02 ± 0.37	0.02 ± 0.01	0.05 ± 0.001
PSB|2 kg/t	7.52 ± 0.25	5.67 ± 0.28	2.87 ± 0.38	28.78 ± 0.81	6.44 ± 0.42	3.15 ± 0.31	0.02 ± 0.01	0.03 ± 0.001
PSB|4 kg/t	7.83 ± 0.21	5.38 ± 0.17	2.90 ± 0.39	28.56 ± 0.78	6.49 ± 0.38	3.16 ± 0.33	0.02 ± 0.01	0.04 ± 0.001
PSB|8 kg/t	7.59 ± 0.24	5.44 ± 0.21	2.92 ± 0.28	28.73 ± 0.82	6.38 ± 0.32	3.32 ± 0.38	0.02 ± 0.01	0.03 ± 0.001

**Table 3:** Final length (FL [mm]), length gain (LG), length-to-weight ratio (W/L [mg/mm]), feed intake (FI [mg/shrimp]), initial live weight of PL30 (IBW [mg]), final body weight of PL65 (FBW [mg]), body weight gain (BWG [mg/shrimp]), feed conversion ratio (FCR [mg/mg]), final weight-to-initial weight ratio (FBW/IBW), and survival (SURV [%]) during the 35 days, from PL30 to PL65 (nursery phase), of shrimp fed increasing levels of protected sodium butyrate (PSB).

PSB (kg/t)	FL	LG	W/L	FI	IBW	FBW	BWG	FCR	FBI/IBW	SURV
PSB|0 kg/t	49.525 ± 0.667^b^	24.363 ± 0.962^b^	14.840 ± 0.362^b^	557.621 ± 34.488	93.009 ± 2.166	735.156 ± 27.327^b^	642.148 ± 28.008^b^	0.870 ± 0.062^a^	7.910 ± 0.389^c^	77.684 ± 2.158^b^
PSB|2 kg/t	51.075 ± 2.371^a^	25.950 ± 2.136^a,b^	16.323 ± 0.701^a^	531.646 ± 25.888	94.365 ± 3.191	834.653 ± 70.089^a^	740.288 ± 70.248^a^	0.721 ± 0.055^b^	8.853 ± 0.821^b^	87.795 ± 0.591^a^
PSB|4 kg/t	51.513 ± 0.881^a^	26.288 ± 1.488^a^	16.418 ± 0.733^a^	539.886 ± 24.903	94.638 ± 1.732	846.031 ± 48.037^a^	751.394 ± 49.048^a^	0.720 ± 0.048^b^	8.946 ± 0.618^b^	87.942 ± 2.644^a^
PSB|8 kg/t	51.550 ± 1.061^a^	26.775 ± 1.812^a^	16.563 ± 0.637^a^	538.563 ± 51.267	92.673 ± 7.986	853.354 ± 18.695^a^	760.681 ± 17.498^a^	0.708 ± 0.070^c^	9.259 ± 0.803^a^	89.415 ± 5.336^a^
*p*-Value	0.0386	0.0658	0.0017	0.6229	0.8539	0.0015	0.0018	0.0005	0.0184	<0.001
SEM	0.552	0.656	0.296	16.332	1.617	20.233	20.401	0.027	0.278	1.524
Linear	0.0041	0.0390	0.0050	0.5770	0.8110	0.0040	0.0040	0.0030	0.0140	<0.001
Quadratic	0.1230	0.2860	0.0620	0.4740	0.4050	0.0440	0.0530	0.0130	0.2840	0.0030
C.V. (%)	2.5000	5.9200	2.3500	6.4900	4.3400	3.8700	6.7100	7.7600	7.7900	3.8000

*Note:* The letters a, b, and c in the columns indicate statistical differences by the Tukey test.

Abbreviation: SEM, standard error of the mean.

**Table 4:** Body weight (BW [g/shrimp]), weight uniformity (UNI [%]), shrimp length (SL [mm]), length uniformity (LU [%]), feed intake (FI [g/shrimp]), body weight gain (BWG [g/shrimp]), feed conversion ratio (FCR [g/g]), length-to-weight ratio (g/mm), final weight-to-initial weight ratio (FBW/IBW), and survival (SURV [%]) during the 80 days after nursery phase (grow-out phase) of shrimp fed increasing levels of protected sodium butyrate (PSB).

PSB (kg/t)	BW	UNI	SL	LU	FI	BWG	FCR	MMG	FBW/IBW	SURV
PSB|0 kg/t	11.196 ± 1.024^c^	79.375 ± 2.066^b^	123.225 ± 3.409^b^	87.125 ± 2.078	18.693 ± 1.076	10.461 ± 1.031^c^	1.804 ± 0.225^a^	11.070 ± 0.817^a^	15.257 ± 1.580^b^	82.231 ± 1.305^b^
PSB|2 kg/t	14.836 ± 0.538^b^	83.250 ± 3.594^a,b^	136.175 ± 1.595^a^	87.850 ± 0.420	17.560 ± 0.214	14.001 ± 0.214^b^	1.255 ± 0.046^b^	9.185 ± 0.223^b^	17.835 ± 1.015^a,b^	86.626 ± 1.024^a^
PSB|4 kg/t	15.884 ± 0.341^a,b^	85.250 ± 4.924^a,b^	137.750 ± 3.307^a^	87.875 ± 0.655	18.101 ± 0.483	15.050 ± 0.483^a,b^	1.204 ± 0.057^b^	8.674 ± 0.213^b,c^	19.182 ± 2.059^a^	88.875 ± 3.023^a^
PSB|8 kg/t	17.239 ± 0.349^a^	86.500 ± 3.000^a^	138.150 ± 4.414^a^	88.125 ± 1.307	17.470 ± 0.612	16.385 ± 0.612^a^	1.067 ± 0.042^b^	8.015 ± 0.249^c^	20.207 ± 0.55^a^	89.410 ± 1.859^a^
*p*-Value	<0.001	0.0087	<0.001	0.6907	0.0642	<0.001	<0.001	<0.001	0.0002	<0.001
SEM	0.3493	1.5172	1.5697	0.7129	0.3708	0.3503	0.07175	0.2651	0.6837	0.8499
Linear	<0.001	0.005	<0.001	0.383	0.08	<0.001	<0.001	<0.001	<0.001	<0.001
Quadratic	<0.001	0.173	<0.001	0.665	0.502	<0.001	0.002	0.003	0.063	0.004
C.V. (%)	5.31	3.92	2.55	0.762	4.38	5.64	10.75	5.90	8.33	2.12

*Note:* The letters a, b, and c in the columns indicate statistical differences by the Tukey test.

Abbreviation: SEM, standard error of the mean.

**Table 5:** The relative weight of the shrimp without head (shrimp[%]), and without all shells (meat[%]) of shrimp meat fed with levels of protected sodium butyrate (PSB).

PSB (kg/t)	Shrimp (%)	Meat (%)
PSB|0 kg/t	59.33 ± 1.3241^b^	47.99 ± 8.6803^b^
PSB|2 kg/t	60.2443 ± 3.2728^a,b^	49.5901 ± 2.7428^a,b^
PSB|4 kg/t	61.4367 ± 2.1428^a^	50.1384 ± 3.2073^a,b^
PSB|8 kg/t	61.7036 ± 2.9178^a^	51.6821 ± 2.6732^a^
*p*-Value	0.0011	0.0442
SEM	0.4616	0.9142
Linear	<0.001	0.006
Quadratic	0.134	0.652
C.V.	4.170	10.05

*Note:* The letters a and b in the columns indicate statistical differences by the Tukey test.

**Table 6:** Hemolymph from shrimp fed with different levels of protected sodium butyrate (PSB): cells per mL of hemolymph from granular, hyaline, and semigranulated cells and the total hemocyte count (THC).

PSB (kg/t)	Granular	Hyaline	Semigranulated	THC
PSB|0 kg/t	2.10 × 10^06^ ± 727,461.34	2.10 × 10^06^ ± 878,493.03	2.86 × 10^06^ ± 1,174,874.46^a^	7.06 × 10^06^ ± 750,996.59
PSB|2 kg/t	2.37 × 10^06^ ± 1,188,886.03	1.51 × 10^06^ ± 654,041.28	1.18 × 10^06^ ± 1,202,696.97^a,b^	5.06 × 10^06^ ± 2,191,977.93
PSB|4 kg/t	1.68 × 10^06^ ± 1,224,499.90	2.44 × 10^06^ ± 1,575,699.84	1.09 × 10^06^ ± 636,961.54^b^	5.21 × 10^06^ ± 2,164,730.00
PSB|8 kg/t	1.68 × 10^06^ ± 1,070,794.10	1.81 × 10^06^ ± 605,004.13	1.26 × 10^06^ ± 742,462.12^a,b^	4.75 × 10^06^ ± 1,868,080.16
*p*-Value	0.687	0.5249	0.0324	0.1881
SEM	4.79 × 10^05^	4.50 × 10^05^	4.35 × 10^05^	7.78 × 10^05^
Linear	0.062	0.241	0.031	0.028
Quadratic	0.793	0.615	0.028	0.015
C.V.	54.72	51.24	60.94	31.55

*Note:* The letters a and b in the columns indicate statistical differences by the Tukey test.

**Table 7:** Shrimp intestine histomorphometry fed with levels of protected sodium butyrate (PSB).

PSB (kg/t)	Villus height (μm)	Width height (μm)	Lamina propria	H:W ratio
PSB|0 kg/t	26.432 ± 13.0734^b^	23.791 ± 12.1846^a^	13.530 ± 8.6486^a^	1.205 ± 0.6216^c^
PSB|2 kg/t	27.690 ± 8.7426^b^	14.623 ± 5.6515^b^	7.205^b^ ± 2.9420^c^	2.097 ± 0.926^b^
PSB|4 kg/t	36.059 ± 9.5246^a^	12.415 ± 3.0418^b^	3.783 ± 1.7274^c^	2.997 ± 0.7984^a^
PSB|8 kg/t	30.229 ± 7.1425^a,b^	21.755 ± 5.8927^a^	9.831 ± 4.9968^a,b^	1.572 ± 0.8342^b,c^
*p*-Value	0.0143	<0.001	<0.001	<0.001
SEM	1.9744	1.368	0.935	0.180
Linear	0.129	0.912	0.89	0.27
Quadratic	0.024	<0.001	<0.001	<0.001
C.V.	32.76	41.27	61.46	40.75

*Note:* The letters a, b, and c in the columns indicate statistical differences by the Tukey test.

## Data Availability

Data will be available upon request from the authors.
